# Which interventions may improve bracing compliance in adolescent idiopathic scoliosis? A systematic review and meta-analysis

**DOI:** 10.1371/journal.pone.0271612

**Published:** 2022-07-20

**Authors:** Xue Li, Zhaohua Huo, Zongshan Hu, Tsz Ping Lam, Jack Chun Yiu Cheng, Vincent Chi-ho Chung, Benjamin Hon Kei Yip

**Affiliations:** 1 School of Public Health and Primary Care, Faculty of Medicine, The Chinese University of Hong Kong, Hong Kong, China; 2 Department of Spine Surgery, Nanjing Drum Tower Hospital of Nanjing University Medical School, Nanjing, China; 3 Department of Orthopaedics and Traumatology, The Chinese University of Hong Kong, Shatin, New Territories, Hong Kong SAR, China; 4 SH Ho Scoliosis Research Laboratory, Faculty of Medicine, The Chinese University of Hong Kong, Shatin, New Territories, Hong Kong SAR, China; 5 Joint Scoliosis Research Center of the Chinese University of Hong Kong and Nanjing University, Hong Kong SAR, China; 6 School of Chinese Medicine, Faculty of Medicine, The Chinese University of Hong Kong, Hong Kong, China; University of Catanzaro: Universita degli Studi Magna Graecia di Catanzaro, ITALY

## Abstract

This review aimed to systematically review and meta-analyze the effects of interventions in improving bracing compliance among adolescent idiopathic scoliosis (AIS) patients. Eight databases were searched from their inception to April 2022. The eligibility criteria included controlled studies that used any type of intervention to enhance bracing compliance in braced AIS patients. Two researchers independently screened articles and extracted data based on the PICO (participant, intervention, comparator, and outcome) framework. Quality appraisal of included studies was performed using GRADE (overall assessment), and the risk of bias was assessed with Cochrane RoB Tool 2 for randomized controlled trials (RCT) and ROBINS-I for non-RCT studies. The primary outcome was bracing compliance and secondary outcomes included Cobb Angle and measurements for quality of life. Six eligible studies involving 523 participants were included. All studies were evaluated as low or very low quality with a high risk of bias. Four types of interventions were identified, including sensor monitoring (n = 2, RCTs), auto-adjusted brace (n = 1, RCT), more intensive or collaborated medical care (n = 2), and psychosocial intervention (n = 1). A meta-analysis of 215 patients from the three RCTs suggested that the compliance-enhancing intervention group had 2.92 more bracing hours per day than the usual care control (95%CI [1.12, 4.72], P = 0.001). In subgroup analysis, sensor monitoring significantly improved bracing wearing quantity compared to usual care (3.47 hours/day, 95%CI [1.48, 5.47], P = 0.001), while other aforementioned interventions did not show a significant superiority. Compliance-enhancing interventions may be favorable in preventing curve progression and promoting quality of life, but the improvements cannot be clarified according to limited evidence. In conclusion, although the results of this study suggested that sensor monitoring may be the most promising approach, limited high-quality evidence precludes reliable conclusions. Future well-designed RCTs are required to confirm the actual benefit of compliance-improving interventions in clinical practice.

## Introduction

Adolescent idiopathic scoliosis (AIS) is the most common form of scoliosis affecting about 1–4% of adolescents [1]. Several long-term disabling complications of curve progression have been noted, including back pain, cardiopulmonary problems, spinal cord injury, and psychosocial concerns, which highlighted the needing for tailored strategies and precise management in AIS treatment [2–5]. Even though few studies suggested that spinal fusion surgery may not generate severe impairment afterward [6, 7], the benefit of surgery is still controversial and more than 500 million dollars per year are spent in the United States alone on AIS surgeries [7, 8]. Thus population-based screening and proper treatment at the onset of disease to prevent curve progression will not only improve AIS patients’ health outcomes but also save healthcare dollars [9–13].

Bracing, which was suggested to be effective in preventing curve progression to surgery threshold [8, 14, 15], is the most commonly used conservative treatment beyond monitoring for AIS patients with an immature skeleton of Cobb angle between 25–45 degrees [3]. To achieve the therapeutic potential of brace treatment, patients’ compliance with bracing is crucial. However, poor compliance with bracing is widely reported in braced AIS patients (33%~77% of prescribed hours) [16–18]. Reasons for non-compliance are associated with the adverse effects of brace treatment, such as the negative cosmetic appearance [19], functional discomfort resulting from pressure points [8], irritation in hot weather [15], and restriction of movement [15]. Emotional discomfort and effects on quality of life are also considered to be important potential psychosocial determinants of compliance [20, 21].

Although the International Society on Scoliosis Orthopaedic and Rehabilitation Treatment (SOSORT) guideline has emphasized the importance of bracing [22], there are currently no clinical guidelines or recommendations on interventions to improve bracing compliance, and there remains a dearth of reviews focusing on the discussion of the behavioral aspects on how to improve post-bracing clinical outcomes. Furthermore, despite that recent studies have reported hypothetical factors that may influence compliance [23], thus far, no systematic review has been conducted to synthesize all existing evidence on the effectiveness of interventions in improving compliance with the bracing regimen. This systematic review aims to fill this knowledge gap by synthesizing the effectiveness of compliance-enhancing interventions for AIS patients.

## Materials and methods

This systematic review was reported following the PRISMA statement for systematic review and meta-analysis [24] (S1 Table).

### Criteria for considering studies for this review

The eligibility criteria were constructed according to the Participants (P), Interventions (I), Comparator (C), and Outcomes (O) (PICO) framework.

#### Study designs

Randomized controlled trials (RCTs), controlled clinical trials without randomization (CCTs) (definition of CCT: investigators had direct control over study conditions but interventions were not randomly assigned [25]), and controlled observational studies (cohort studies, and case-control studies) were included because it was anticipated that very few RCTs would be identified.

#### Population

We included studies in which all patients, following a confirmed clinical and radiological diagnosis of AIS, were prescribed bracing treatment.

#### Interventions and comparators

The experimental interventions in this review included all types of interventions that were considered to aim at improving bracing compliance. Controls were usual care of bracing treatment without any component to increase bracing compliance.

#### Outcomes

The primary outcome is bracing compliance, as measured by the percentage of prescribed regimen, the number of daily bracing hours, or the proportion of compliant patients [8, 26]. Binary outcome variables (good compliance vs poor compliance) were presented as risk ratio (RR) or odds ratio (OR), and continuous outcomes were presented as means, standard deviations (SD), and/or standard errors (SE). We did not set the restriction on the assessment method of bracing wearing compliance (i.e., self-report or sensor records).

Secondary outcomes are scoliosis parameters, as measured by Cobb angle in degrees, angle of trunk rotation (ATR) in degrees, the number of patients who have progressed by more than 5° Cobb, or the number of subjects for whom surgery was prescribed. Quality of life data, as measured by specific validated questionnaires, e.g. SRS-22, SF-36 [27], BrQ [28] was also extracted.

### Search methods for identification of studies

**Fig 1:**
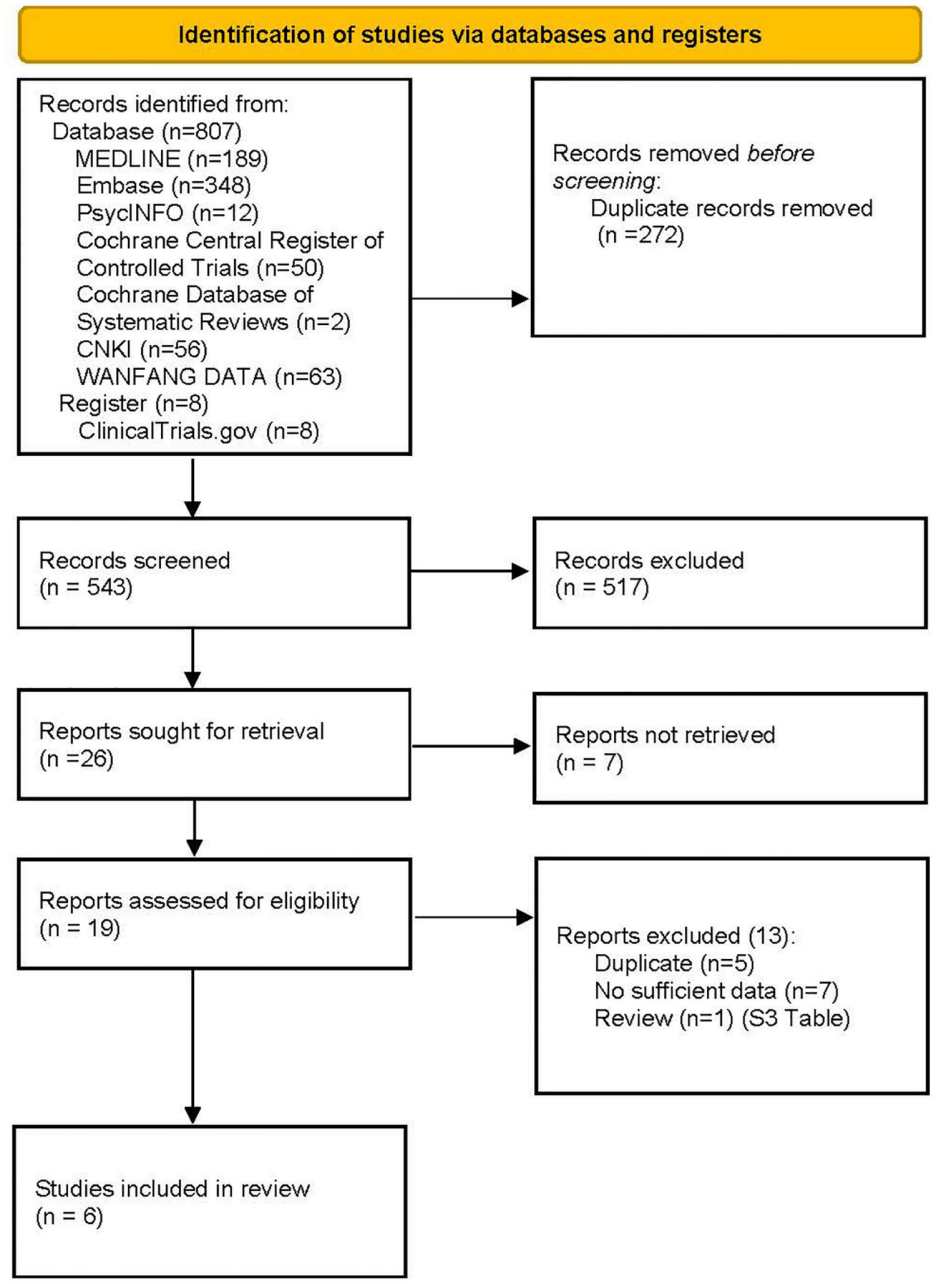
PRISMA 2020 flow diagram demonstrating the literature search and screening process.

Online literature searches were performed on the six international databases and two Chinese databases. The starting year is the inception for each database, e.g. 1946 for Medline, and the search was conducted in April 2022. The terms of participants (AIS patients) and primary outcome (bracing compliance) were intentionally applied to achieve a comprehensive retrieval of records from databases without the restriction of publication status (Fig 1). The search strategy for each database is detailed in the S2 Table in the appendix. The reference lists of relevant reviews were scrutinized for further articles. Searching of the main online sources of ongoing trials (National Research Register, meta-Register of Controlled Trials; Clinical Trials) for available data was conducted, including grey literature, including conference proceedings and Ph.D. theses. Authors of registered trials were contacted for possible available data to identify any further studies we could include.

### Data collection and analysis

#### Selection of studies

Two review authors (XL, ZSH) independently screened the search results based on titles and abstracts, followed by an independent full-text assessment of potentially relevant studies. The pre-specified inclusion and exclusion criteria of eligibility which were drafted according to the PICO framework were adopted for studies selection. Disagreements between the two authors were resolved by discussion until consensus was reached or through discussion with a third researcher (BHKY).

#### Data extraction and management

The data of the included papers were extracted in predesigned data extraction form by two independent reviewers (XL and ZHH). Extracted variables included: (1) study characteristics (study design, country, year, recruitment modality, risk of bias); (2) patient characteristics (number of participants, age, sex, baseline compliance); and (3) description of the experimental and comparison interventions, co-interventions, adverse effects, duration of follow-up, outcomes assessed, and results. Disagreements were resolved through discussion. Key findings were summarized in a narrative format and then assessed for inclusion in a meta-analysis where possible.

#### Assessment of risk of bias and methodological quality

We assessed the risk of bias in RCTs in this review using the Cochrane risk-of-bias tool for randomized trials (RoB 2) [29] according to the following domains: bias arising from the randomization process; bias due to deviations from intended interventions; bias due to missing outcome data; bias in the measurement of the outcome; and bias in the selection of the reported result. Observational studies and CCTs were assessed using the Risk Of Bias In Non-randomised Studies-of Interventions (ROBINS-I) tool [30, 31]. The ROBINS-I assesses four broad areas: confounding, selection bias, information bias, and reporting biases.

The overall quality of the evidence for the primary outcome was assessed with the adapted GRADE approach [32, 33]. Domains that may decrease the quality of the evidence are study design and implementation (risk of bias), inconsistency (heterogeneity), indirectness (inability to generalize), imprecision (insufficient or imprecise data), and publication bias across all studies that measure that particular outcome. The quality of the evidence on a specific outcome is based on the performance against six factors: study design, risk of bias, consistency, and directness of results, the precision of the data, and publication bias across all studies that measured that particular outcome.

Two reviewers (XL, ZHH) appraised each study independently and disagreements were resolved through discussion with a third reviewer (BY).

### Data synthesis

The primary analysis was comparisons of compliance-enhancing interventions versus no intervention or other interventions for two or more studies with the same study design. Standard deviations were calculated for meta-analyses purposes if they weren’t provided. The mean differences (MD) with 95% confidence intervals were calculated for all continuous variables. Multiple subgroups classified according to the dose of interventions were combined into a single intervention group [34]. In subgroup analysis, pooled estimates were conducted where two or more studies were adopting a similar intervention. The random-effects inverse variance model was used due to the possible variation in study methodology and bracing regimen applied (brace type, recommended wearing hours). Heterogeneity was assessed using the I^2^ value. Review Manager Software, version 5.3 was used for the analysis.

## Results

### Results of the search

From the bibliographic search, we identified 807 references. After removing duplicates, we identified 543 potentially relevant references; 517 were excluded based on title and abstracts, leaving 26 studies that were acquired in full text or study report with available information for further evaluation. (Fig 1). After conducting a full-text review, six studies [35–40] were included in our systematic review. A hand-search of references of the included studies revealed no further relevant publications. Substantive descriptions of the included studies can be seen in Table 1, while the reasons for excluding studies after full-text review are listed in S3 Table.

**Table 1:** Characteristics of included studies.

First author, year, district	Study design	Inclusion criteria	No. of participants (I, C ^a^)	Gender (% of female)	Intervention	Comparator	Bracing prescription (hours/day)
*Al-Aubaidi, 2013, Denmark*	Controlled clinical trial	Bracing AIS patients, Risser 0–3	I: 12, C:12	I:100%, C:100%	Treatment initiated during hospitalization for 2–3 days	Outpatient clinic two weeks after the brace was delivered	≧8
*Karol, 2016, USA*	Randomized controlled trial.	AIS patients Risser stage 0–2; and, if female, less than one-year post menarche.	I: 93, C:78	I: 88%, C: 92%	Be informed of the installment of sensors monitoring bracing compliance and be counseled regarding the bracing compliance report in follow-up.	Be told that sensors monitoring temperature rather than compliance and received usual care	NA
*Lin, 2020, Hong kong*	Randomized controlled trial	Females AIS patients, age 10 to 14, Risser sign 0 to 2, Cobb 20° to 40°, pre-menarche or within 24 months after menarche.	I: 11, C:12	I:100%, C:100%	Automated pressure-adjustable orthosis	Conventional rigid orthosis	23
*Miller, 2012, USA*	Randomized controlled trial	AIS patients aged 8 to 15 years, previously untreated, skeletally immature, and willing to undergo brace treatment	I:10, C:11	I:70%, C:82%	Be informed that their compliance was monitored before treatment.	Be not informed placement of a compliance monitor before treatment.	18
*Negrini, 2014, Italy*	Retrospective controlled cohort study	AIS patients with first brace prescription and regular use of Thermobrace heat sensor; two evaluations after bracing; age >6; European Risser 0–3.	I:143, P: 52; C: 51 ^b^;	NA	Cognitive Behavioural Approach (CBA) dispensed during Physiotherapic Scoliosis Specific Exercises (PSSE) sessions in 0–4 month	No intervention	21.93±1.77;
*Tavernaro, 2012, Italy*	Retrospective case-control study	AIS or hyperkyphosis patients (10 years or more) in the brace for at least 6 months with at least 15 hours/day of brace wearing:	I: 13, C: 25;	I:77%, C: 58%	Treated by a complete team where physiotherapists served as the main aggregator of the whole team in the private institute	Treated in a team with weak connections between physician/orthotist and the physiotherapists in Rehabilitation Department of the Italian Health National Service (HNS).	I:17.2 ± 3.6; C:17.7 ± 4.1

^a^ I: intervention group; C: control group

^b^ I: group with good compliance to intervention; P: group with poor compliance to intervention; C: control group without intervention

### Included studies

The six included studies were published between 2012 and 2020. They consisted of three RCTs [35, 36, 40], one non-randomized controlled trial [37], one retrospective cohort study [39], and one retrospective case-control study [38] (Table 1). The median sample size was 31 participants (range: 21–246 participants). Two studies were conducted in the USA [35, 36], two in Italy [38, 39], one in Denmark [37], and one in Hong Kong [40]. The mean age of participants across 6 studies ranged from 11.9 to 15.8 years and the proportion of girls in each study ranged from 68.4% to 100%. Bracing compliance was assessed through self-reporting [37, 38] and sensor monitoring [35, 36, 39, 40].

### Risk of bias and quality assessment

**Fig 2:**
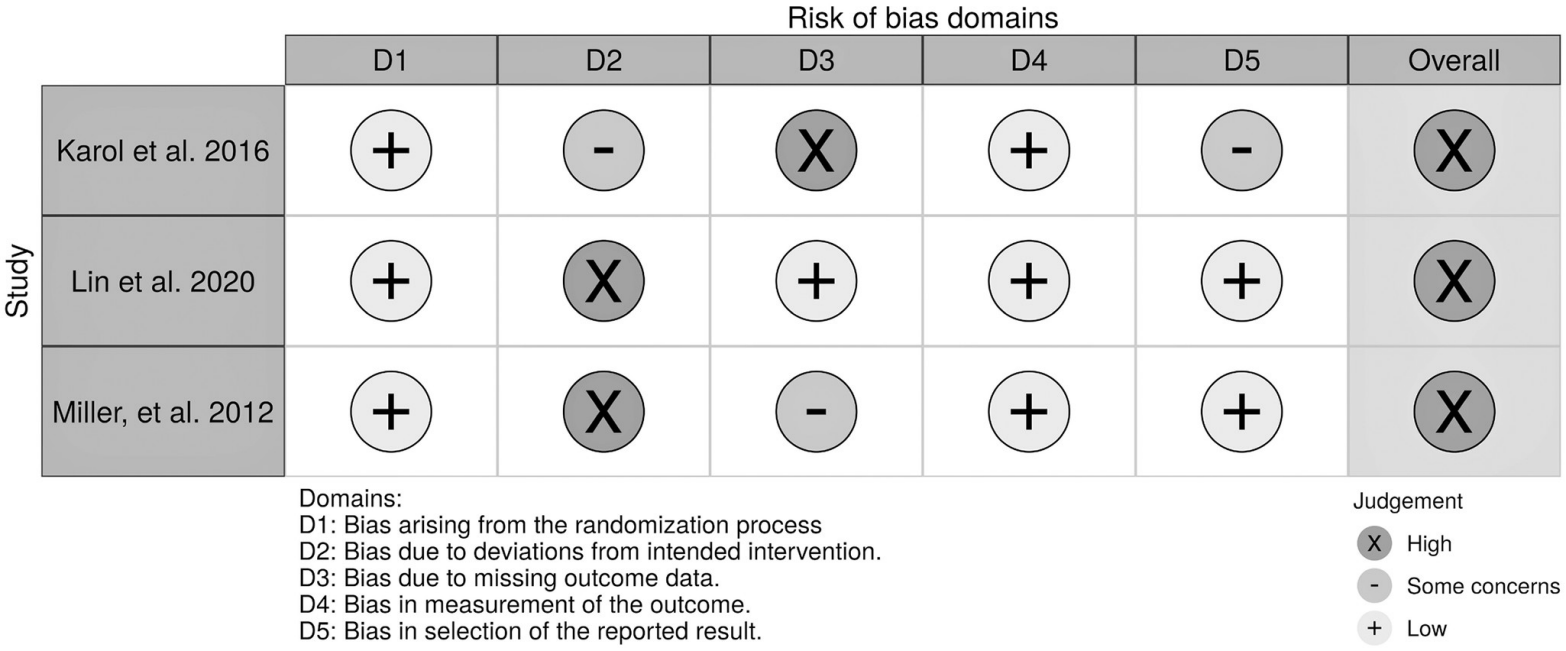
Judgement of risk of bias which was assessed according to the Cochrane’s risk of bias tool V2 for RCTs.

**Fig 3:**
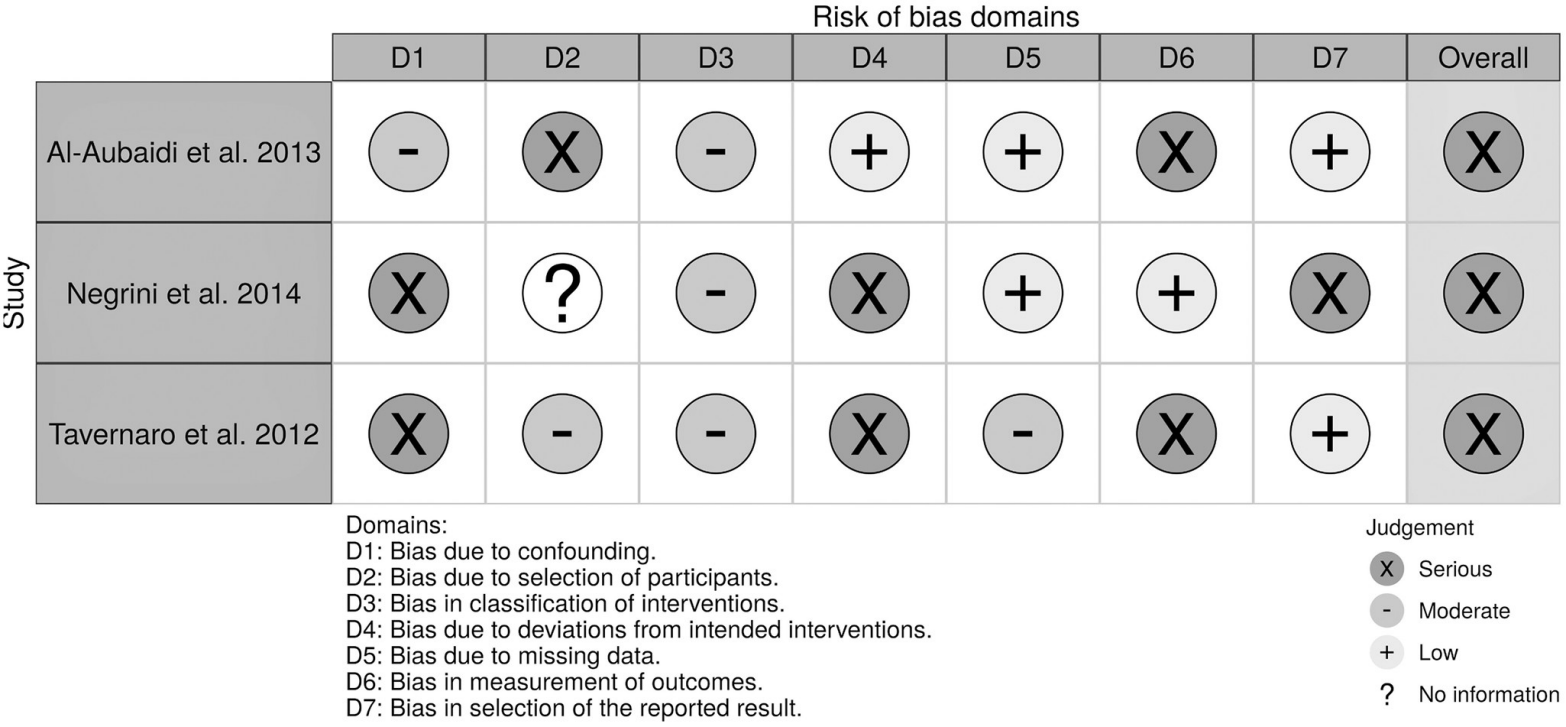
Judgement of risk of bias which was assessed using the Risk OF Bias in Non-randomized Studies-of Interventions (ROBINS-I) tool for observational studies.

The overall risk of bias was high for three RCTs (Fig 2), and in particular, there were some concerns or high risk of bias due to missing outcome data and some concerns or high risk of bias due to following modified intention -to treat principle. In Karol et al study [41], only patients with complete data were included in the final analysis, which increased the risk of bias toward favoring the intervention. In Miller et al. trial [35], the severe drop-out rate and the limited sample size are also a worry. Furthermore, none of the RCTs collected baseline compliance data and as a consequence not be able to adjust it in their analyses to prevent potential regression-to-mean results. Fig 3 illustrates the results of the risk of bias assessment of the observational studies and non-randomized controlled studies. Overall, all three studies suffer from a high risk of bias, particularly bias due to confounding and the measurement of outcomes. Details of the quality ratings of GRADE are presented in the S4 Table.

### Interventions

Sensor monitoring [35, 36], psychosocial intervention [39], interventions of the change of medical care [37, 38], and auto-adjusted brace [40] were identified in included studies (Table 1).

Two RCTs [35, 36] investigated sensor monitoring vs. not monitoring. Patients in the intervention group were monitored using electronic sensors, which were installed on the braces and consisted of a temperature probe and can store compliance data measured by temperature [35]. In addition to an embedded temperature sensor, Karol et al. [36] provided patients with feedback counseling according to records retrieved from sensors at clinical visits, while standard clinical service was provided to the control group.

In a non-randomized trial, Al-Aubaidi et al. [37] compared an intensive hospitalization approach, which provided more prompt adjustments for participants for bracing adaptation in a few days, with a less intensive approach that was conducted in an outpatient clinic. Noteworthy, in Denmark, the intensive hospitalization approach is the usual practice, while the outpatient clinic approach was the intervention. This is different from other countries, at least those that were included in this systematic review, where outpatient service is more widely used as usual care for braces adaption and adjustment in scoliosis clinical practice. Using a retrospective case-control design, Tavernaro et al. evaluated the compliance-enhancing effect of a complete, multi-professional expert rehabilitation team [38], which involved parents and patients, collaborating closely through the aggregation of physiotherapists.

In a cohort study, the effect of the Cognitive Behavioural Approach dispensed during Physiotherapic Scoliosis Specific Exercises (CBA-PSSE) was studied [39]. Bracing compliance (0–4 months) was compared among three patient groups with different levels of adherence to CBA-PSSE intervention (good adherence to the intervention group (I):≧2 sessions, poor adherence group (P):1 session, control group (C): 0 sessions).

One RCT [40] was found to investigate the effectiveness of a newly developed automated pressure-adjustable orthosis, which could maintain a more consistent interfacial corrective effect at the prescribed level by inflation and deflation of the air bladder. Compliance monitoring sensors of similar size compared to the smart device for the intervention group were installed in the same area in the conventional rigid braces for the control group.

### Intervention effects on bracing compliance

**Fig 4:**
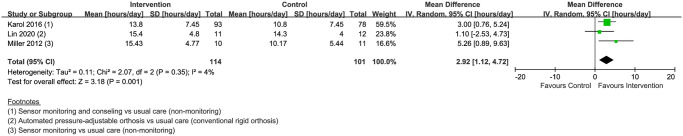
Forest plot of mean differences (with 95% confidence intervals) and study weights for three bracing compliance enhancing RCT studies.

The results of the effects on bracing compliance for included studies are summarized in Table 2. The patients in groups with good (number of attending sessions>1) (I) and poor (number of attending sessions = 1) (P) adherence to the CBA intervention in the Negrini et al. study [39], were combined into a single intervention group in the meta-analysis. To be comparable with other studies, we treated outpatient clinics in Al-Aubaidi et al. study [37] as the control group in the meta-analysis. Three studies reported average wearing hours per day [35, 36, 40], while five studies reported the percentage of prescription wearing time as the outcome was reported or could be calculated [35, 37–40]. Using the former as the outcome measure, a meta-analysis was conducted with 215 patients from three RCTs [35, 36, 40] (intervention: n = 114, control: n = 101), and it indicated that higher bracing compliance can be achieved through interventions, i.e., the interventions group had on average 2.92 more bracing hours per day (95%CI [1.12, 4.72], P = 0.001). Low inconsistency was found in effect size (P = 0.35, I^2^ = 4%). (Fig 4).

**Table 2:** Intervention effect on bracing compliance.

Study	Intervention	Outcome definition	Assessment of compliance	Assessment points	Intervention (Mean/SD)	Control (Mean/SD)	Effect size (MD/OR 95% CI)	P-value	Quality of evidence (GRADE)
**Al-Aubaidi 2013**	Outpatient service	Percentage of prescription wearing time (%)	Self-report	≧3 months	89% (17.31%)	81% (20.46%)	8.00% (-7.16, 23.16)	0.312	⨁◯◯◯ Very low
**Tavernaro 2012**	Team approach	Proportion of compliant patients (total wearing time≧90% prescription)	Self-report	I:1.5 ± 0.5 years, C:1.2 ± 0.4 years	NA	NA	5.5 (3.6, 7.4) ^b^	<0.05	⨁◯◯◯ Very low
		Percentage of prescription wearing time (%)	Self-report	I:1.5 ± 0.5 years, C:1.2 ± 0.4 years	97% (6%)	80% (24%)	17% (5.05, 28.95)	0.030	
**Karol 2016**	Sensor monitoring,	Average wearing hours per day (hours)	Thermochron iButtons sensor	6 months	15.0 h/day (NA)	12.5 h/day (NA)	2.50 h/day (0.63, 4.37)	0.0095	⨁⨁◯◯ Low
		Average wearing hours per day (hours)	Thermochron iButtons sensor	Entire brace treatment	13.8 h/day (7.45 h/day)	10.8 h/day (7.45 h/day)	3.00 h/day (0.76, 5.24)	0.002	
**Miller 2012**	Sensor monitoring	Percentage of prescription wearing time (%)	the StowAway TidbiT temperature monitor	3.5 months	85.7% (26.5%)	56.5% (30.2%)	31.30% (5.12, 57.48)	0.029	⨁⨁◯◯ Low
		Average wearing hours per day (hours)	the StowAway TidbiT temperature monitor	3.5 months	15.43 h/day (4.77 h/day)	10.17 h/day (5.44 h/day)	5.26 h/day (0.89, 9.63)	0.030	
**Lin 2020**	Automated pressure-adjustable orthosis	Percentage of prescription wearing time (%)	Temperature sensor	1 year	66.96% (20.87%)	62.17% (17.39%)	4.79% (-10.99, 20.57)	0.55	⨁⨁◯◯ Low
		Average wearing hours per day (hours)	Temperature sensor	1 year	15.4 h/day (4.8h/day)	14.3 h/day (4.0 h/day)	1.10 h/day (-2.53, 4.73)	0.55	
**Negrini 2014**	Psychosocial intervention	Percentage of prescription wearing time (%)	Thermobrace heat sensor	4 months	I ^a^: 90.63% (11.95%) P ^a^ 93.62% (10.89%)	C ^b^:89.66% (15.68%)	I vs C: 0.97% (-3.76, 5.70)	0.648	⨁◯◯◯ Very low
							P vs C: 3.96% (-1.26, 9.18)	0.139	

^a^ I: group with good compliance(number of attending sessions>1) to CBA+PSSE intervention during 0–4 months after brace delivery; P: group with poor compliance(number of attending sessions = 1) to CBA+PSSE intervention during 0–4 months; C: control group without attending CBA+PSSE intervention(number of attending sessions = 0) during 0–4 month;

^b^ Except for Tavernaro et al. study which reported an odds ratio for the outcome of the proportion of compliant patients (total wearing time≧90% prescription), the effect size of all studies was reported using mean difference and 95% confidence interval.

#### Sensor monitoring vs non-monitoring

**Fig 5:**
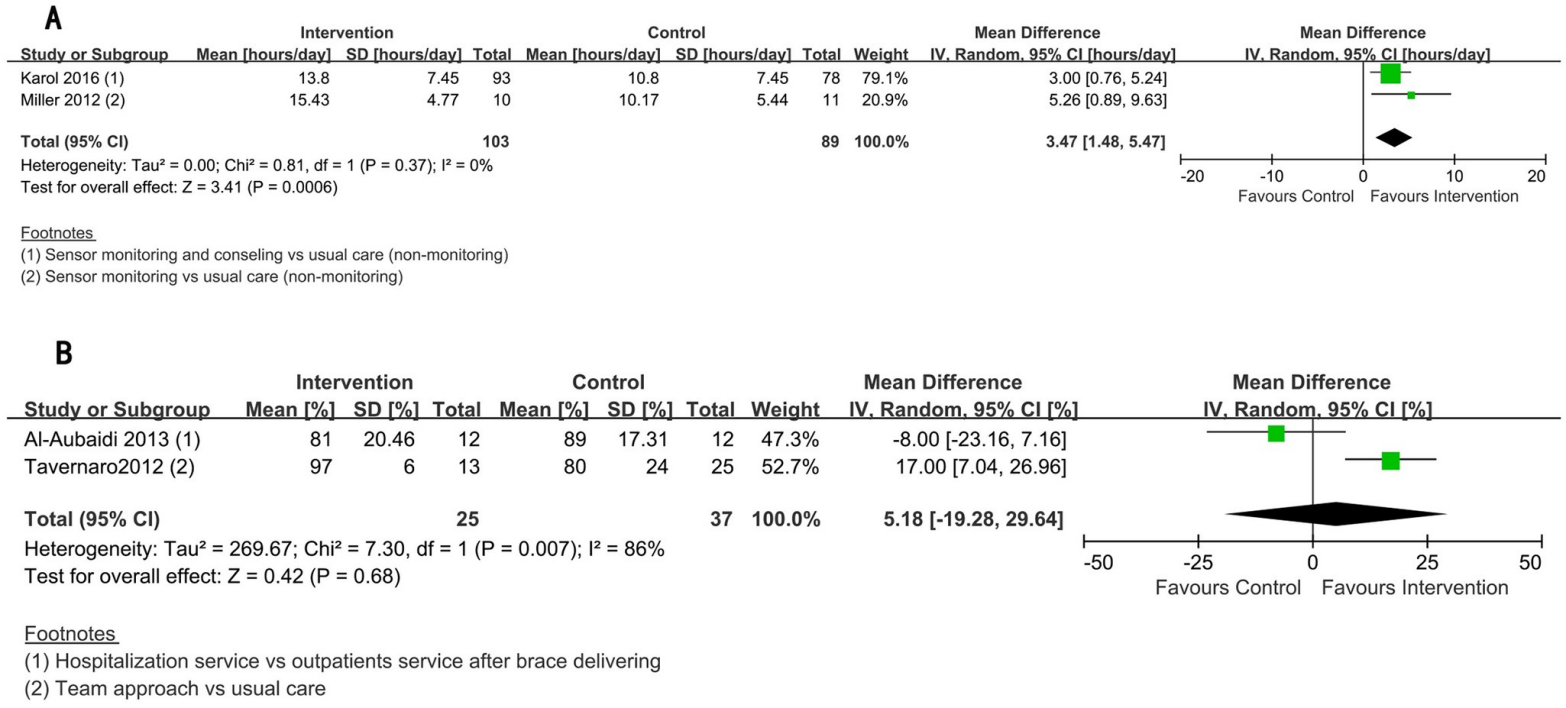
Forest plot of mean differences (with 95% confidence intervals) and study weights for studies with similar interventions (sensor monitoring intervention, and medical care intervention).

Two RCTs which consisted of 192 participants, reported average bracing hours per day as the outcome [35, 36]. The meta-analysis revealed that bracing compliance significantly improved with monitoring when compared to no monitoring (3.47 hours/day, 95%CI [1.48, 5.47], P < 0.001). There was no evidence of heterogeneity (P = 0.37, I^2^ = 0%) (Fig 5A).

#### Intervention of medical care vs usual care

Compliance data, which was defined as the percentage of the prescription wearing time, from 2 studies [37, 38] and 62 participants were available (Tables 1 and 2). The results favored the change in medical care, but the improvement in compliance was not significant (5.18%, 95% CI [−19.28, 29.64], P = 0.68) (Fig 5B). High inconsistency in effect size was observed (P = 0.007, I^2^ = 86%). For the result in single studies, the percentage (MD = 17%, 95%CI [5.05, 28.95], P = 0.03) and the odds ratio of achieving good compliance (OR = 5.5, 95%CI [3.6, 7.4], P<0.05) were reported to be significantly higher for a collaborated medical team approach as compared with usual care [38], while no superiority was detected for the hospitalization intervention with more intensive medical care [37].

#### Psychosocial intervention vs no intervention

For the Cognitive Behavioural Approach versus no intervention comparison, data from only one study was available (Table 2). The percentage of prescription wearing time favored of intervention groups more than the control but did not reach the significance (I vs C: MD = 0.97%, 95%CI [-3.76, 5.70]; P vs C: MD = 3.96%, 95%CI [-1.26, 9.18]) (Table 2). The combined intervention group also did not present a significant superiority compared with the control (MD = 1.77%, 95%CI [-2.84, 6.38]).

#### Auto-adjusted brace vs usual care (conventional orthosis)

In the automated pressure-adjustable brace versus conventional rigid orthosis analysis, there was only one RCT [40] with 23 participants where data on bracing compliance was available. There was no significant difference between the effect of the new brace and the conventional brace (MD = 1.10 h/day, 95%CI [-2.53, 4.73], P = 0.55). (Table 2, Fig 4).

### Radiographic outcome

The rate of bracing success (curves progression< 6°) was higher in the sensor monitored group than that in the non-intervention group (55/93 vs 36/78, RR 1.28, 95% CI 0.96–1.72, P = 0.098), and quite the opposite for the rate of failure (progression to cobb angel> 50°or surgery) (23/93 vs 28/78, RR 0.69, 95% CI 0.43–1.09, P = 0.114) [36]. Cobb angle data from one trial [40] revealed that at 1-year follow-up 4 out of 11 patients who were treated with an auto-adjusted brace had a curve reduction of more than 5°; while the number was 2 out of 12 in the control group with rigid braces (P = 0.156). The results of radiographic outcomes favor interventions in both studies although not significantly so.

### Quality of life

A better level of quality of life measured by SRS-22 was found to be promoted by team approach [38] (4.13±0.46 vs 3.39±0.60, MD = 0.74, 95%CI [0.26, 1.22], P = 0.01), but not by auto-adjusted brace [40] (4.3±0.2 vs 4.3±0.4, MD = 0.00, 95%CI [-0.26, 0.26], P = 1.00). There was no significant difference concerning the score of the scoliosis Quality of Life Index (SQLI) questionnaire [42] between the outpatient service contrast the hospitalization in the Al-Aubaidi et al. study [37] (median = 77, IQR:73–87 vs median = 78, IQR:69–88).

## Discussion

This is the first review that has systematically analyzed the effectiveness of compliance-enhancing interventions in braced AIS patients. We identified four approaches that were studied in the AIS population, including sensor monitoring [35, 36], more intense or collaborated medical care [37, 38], psychosocial intervention [39], and auto-adjusted brace [40]. Among the identified interventions, sensor monitoring may be the most promising approach. Interventions may be favorable concerning the effect of preventing curve progression [36] and promoting an improved quality of life [37, 38]. However, the clinical importance of the improvements still cannot be clarified according to limited evidence and limitations in the methodology quality of current studies.

In this study, electronic monitoring is considered to be most promising given its effect size on bracing compliance improvement and it has been demonstrated to be effective in improving medication adherence for patients with chronic diseases [43–46]. More optimal compliance could be observed when patients were informed to be objectively monitored, which may be a good use of the Hawthorne effect [47, 48]. Besides, as an accurate assessment of brace wearing is the basic necessary information when we try to improve bracing compliance, objective sensor monitoring should be considered as the routine method in the management of braced patients with AIS in clinical practice to ensure the effect of bracing treatment.

The positive influence of the integrated clinical team approach is also in line with evidence supporting the use of innovative, modified health care teams in enhancing patients’ compliance rather than traditional, independent physician practice and minimally structured systems [49, 50]. The underlining reason could be that good communication among patients and health professionals may contribute to a better flow of important clinical and psychosocial information, building trust, and providing support [51].

AIS patients may experience issues of low self-esteem, body image, social role definition, and stress [52], which may cause them to rebel against the regimen [53]. Therefore, although the certainty of the evidence is limited for confirming the effectiveness of CBT+PSSE intervention with meta-analysis in this review, it is still a considerable attempt to involve social behavior theory models (e.g., social cognitive theory self-regulation model, and social support theory) in the development of future effective interventions, since the wide application on treatment adherence in other diseases [54–57].

According to previous evidence, bracing compliance of AIS patients can be affected by age [58], gender [59], BMI index [60], the type of braces (structure and appearance) [15, 61–64], and brace wear pattern (daytime/ nighttime or part-time/ full-time) [65, 66]. As the core device of brace treatment, the optimal brace is the primary consideration. Braces promote proper spinal growth and motor behaviors by reducing unnatural loading and asymmetrical movements, which can be brought about by mechanical forces and external and proprioceptive inputs [67, 68]. Adequate wearing quantity could contribute to orthotic treatment effectiveness [8, 69]. Compared with traditional rigid spinal orthosis (e.g., Boston brace), elastic or flexible braces, in which movement is only partially restricted, were suggested as being more acceptable but with considerably higher rates of curve progression [15, 17, 70, 71]. Orthosis stabilization power and patient experience need to be balanced to achieve the best in quality and quantity of brace wearing. The relative efficacy of different types of braces on bracing compliance cannot be confirmed in this review, due to only one study being identified that provided comparative data. Compliance data for different types of braces need to be further collected and compared in future studies.

Only one of the included studies examines radiographic outcomes (e.g., cobb curve progression) [36], and 2 studies evaluated patient-relevant outcomes (e.g., quality of life) [37, 38]. Given the limited number and quality of studies, an evaluation of the actual benefit of the compliance-improving interventions is difficult. Previous evidence suggested that poor bracing compliance is associated with poorer quality of life and a higher risk of progression [8, 72]. Additional well-controlled research establishing whether the current findings generalize to bracing compliance, patient-related outcomes, and clinical outcomes is important for establishing the viability of hypnosis as an effective intervention.

### Limitation

Although promising insights have emerged from this review, the current findings have several important limitations. The largest challenge is that only 6 eligible studies have focused on this topic. The lack of studies makes it difficult to analysis by each sub-intervention and derive a definitive reliable conclusion. Given high heterogeneity in methodologies, the included studies are difficult to be compared directly. There are differences in regimens applied (recommended wearing hours) and baseline characteristics (age and gender) across the included studies which may influence adherence [23]. Furthermore, for self-reporting assessments, which were adopted by two included studies [37, 38], a higher estimation of intake rather than the true adherence rate has been shown [73]. Objective assessment methods (e.g., electronic temperature monitoring) need to be considered in future studies. In addition, the possible confounding factors of environmental temperature and unfit braces should be noted in the objective assessment. Besides, the follow-up time of three out of six studies was shorter than 1.5 years [35, 37, 39], and only Karol et al. study covered the entire bracing treatment period [36]. It is still uncertain whether the intervention effect was sustained throughout the long treatment period, which consists of an average of 2.5 years of bracing [1]. Future studies might incorporate a longer follow-up period to clinical endpoints into their study designs while being mindful of the greater attrition rates with increased study lengths.

The methodological quality of the included trials also needs to be considered when interpreting these results. Firstly, only one study [37] included sample size calculations to detect statistically significant differences in their methodology. Adequate power is needed to reduce the risk of random error and false-positive results [74]. Secondly, in all four included trials [35–37, 40], the data were not analyzed according to intention-to-treat principles. This criterion is a source of bias because it could be assumed that patient groups that are at risk of being nonadherent are also more likely to be lost to follow-up. Thirdly, 5 studies reported without a baseline assessment of compliance outcomes [35–39] and all of the included studies did not conduct difference in difference analysis to evaluate the effectiveness of interventions [75]. The effect of interventions can be overestimated or underestimated when the sample size is limited. The likelihood of finding only slight differences between groups can be increased by a high baseline adherence level, which may result in a ceiling effect that limits room for improvement and a marginal group effect.

### Impact on future practice and research

The modest effect sizes found in this review demonstrate the difficulty in changing compliant behavior which was also reported in the management of other diseases [76, 77]. In the future intervention design, clinicians need to consider the challenges in changing adherence behavior and make this a priority. Other add-on medical care, such as Schroth physiotherapeutic exercises, also can be recommended to individuals who are not compliant with bracing treatment as compensation for the standard treatment [78]. Furthermore, we are making the following recommendations for future brace compliance intervention trials in their study design to address the aforementioned limitations and heterogeneity we found in this systematic review. Firstly, a standardized objective assessment of bracing compliance (e.g. sensor monitoring) is essential for an accurate evaluation of the intervention effect. Secondly, to avoid regression-to-mean findings, due to an imbalance in baseline bracing measure, we strongly recommend that future RCTs should collect baseline compliance data (using the objective measure mentioned above) and adjust it by using the analysis of covariance (ANCOVA) principle, which is the preferred method for RCT when the outcome is continuous. ANCOVA also provides a greater statistical power to detect a true treatment effect than other approaches (post-only, pre vs post, or percentage change) [79, 80]. Furthermore, the follow-up time of all identified studies is limited to assess the sustainability of the intervention and its impact on clinical outcomes, e.g., curve progression. A longer follow-up time designed according to the 2.5 years of bracing treatment time on average could be considered [1]. Lastly, there are likely other factors that were not captured in these intervention techniques and could impact adolescents’ decision to wear their braces or not. Few interventions included in this review have attempted to tailor intervention approaches to patients’ barriers to good bracing compliance, e.g., negative cosmetic appearance [19], discomfort, and restriction of braces resulting from pressure points [8, 15], emotional problems, or poor quality of life [20, 21]. Future interventions can be designed using multiple strategies targeting these barriers to increase the likelihood of addressing the reasons for non-compliant for any given AIS patient.

## Conclusion

Interventions of sensor monitoring, more intense or collaborated medical care, psychosocial intervention, and auto-adjusted brace have been studied for improving bracing compliance in AIS patients. Of these, sensor monitoring may be the most promising approach. The evidence is, however, not conclusive due to various limitations. We recommend future randomized controlled trials of bracing compliance intervention to have an adequate sample size, with longer follow-up to clinically relevant endpoints, and using objective measurements of compliance outcomes at baseline and post-intervention.

## Supporting information

S1 Table Preferred reporting items for systematic reviews and meta-analysis (PRISMA) checklist. (DOCX)

S2 Table Full search strategies. (DOCX)

S3 Table List of excluded studies and reasons for exclusion. (DOCX)

S4 Table Overall GRADE quality assessment for the primary outcome (bracing compliance). (DOCX)
