# Supplementary material for: Which interventions may improve bracing compliance in adolescent idiopathic scoliosis? A systematic review and meta-analysis
Source: PLoS One. 2022 Jul 20;17(7):e0271612. doi: 10.1371/journal.pone.0271612 (PMC9299303; doi:10.1371/journal.pone.0271612)
Supplement: S2 Table — (DOCX) [file pone.0271612.s002.docx]

**S2 Table Full search strategies**

| **Database (provider)** | **Search strategy** |
| --- | --- |
| ***EMBASE (via ovid),*** | 1. scoliosis.ti. or scoliosis.ab. or scoliosis.sh.  2. AIS.ti. or AIS.ab. or AIS.sh.  3. adolescent.ti. or adolescent.ab. or adolescent.sh.  4. child.ti. or child.ab. or child.sh.  5. children.ti. or children.ab. or children.sh.  6. adherence.ti. or adherence.ab. or adherence.sh.  7. compliance.ti. or compliance.ab. or compliance.sh.  8. 1 or 2  9. 3 or 4 or 5  10. 6 or 7  11. 8 and 9 and 10  (370 – citations retrieved) April 2022 |
| ***MEDLINE (via ovid)*** | 1. scoliosis.ti. or scoliosis.ab. or scoliosis.sh.  2. AIS.ti. or AIS.ab. or AIS.sh.  3. adolescent.ti. or adolescent.ab. or adolescent.sh.  4. child.ti. or child.ab. or child.sh.  5. children.ti. or children.ab. or children.sh.  6. adherence.ti. or adherence.ab. or adherence.sh.  7. compliance.ti. or compliance.ab. or compliance.sh.  8. 1 or 2  9. 3 or 4 or 5  10. 6 or 7  11. 8 and 9 and 10  (225 – citations retrieved) April 2022 |
| ***PsycINFO (via ovid)*** | 1. scoliosis.ti. or scoliosis.ab. or scoliosis.sh.  2. AIS.ti. or AIS.ab. or AIS.sh.  3. adolescent.ti. or adolescent.ab. or adolescent.sh.  4. child.ti. or child.ab. or child.sh.  5. children.ti. or children.ab. or children.sh.  6. adherence.ti. or adherence.ab. or adherence.sh.  7. compliance.ti. or compliance.ab. or compliance.sh.  8. 1 or 2  9. 3 or 4 or 5  10. 6 or 7  11. 8 and 9 and 10  (12 – citations retrieved) April 2022 |
| ***Cochrane Central Register of Controlled Trials (CENTRAL)(via Chocharance liabrary)*** | 1. (scoliosis):ti,ab,kw OR (AIS):ti,ab,kw  2. (adolescent):ti,ab,kw OR (child):ti,ab,kw OR (children):ti,ab,kw  3. (adherence):ti,ab,kw OR (compliance):ti,ab,kw  4. #1 AND #2 AND #3  (79 – citations retrieved) April 2022 |
| ***Cochrane Database of Systematic Reviews*** | 1. (scoliosis):ti,ab,kw OR (AIS):ti,ab,kw  2. (adolescent):ti,ab,kw OR (child):ti,ab,kw OR (children):ti,ab,kw  3. (adherence):ti,ab,kw OR (compliance):ti,ab,kw  4. #1 AND #2 AND #3  (2 – citations retrieved) April 2022 |
| ***ClinicalTrials.gov*** *(*[*http://clinicaltrials.gov/ct2/search/advanced*](http://clinicaltrials.gov/ct2/search/advanced)*)* | (“compliance” OR “adherence”) AND ( “scoliosis” OR “AIS”)  (8 – citations retrieved) April 2020 |
| ***CNKI (via CNKI)*** | (KY=Compliance OR KY=Adherence OR KY=’依从性’ OR KY=’佩戴时长’ OR TI=Compliance OR TI=Adherence OR TI=’依从性’ OR TI=’佩戴时长’ OR AB=Compliance OR AB=’依从性’ OR AB=’佩戴时长’) AND (KY='脊柱侧弯' OR KY='特发性脊柱侧弯' OR KY=scoliosis OR TI='脊柱侧弯' OR TI='特发性脊柱侧弯' OR TI=Scoliosis OR AB='脊柱侧弯' OR AB='特发性脊柱侧弯' OR AB=Scoliosis)  (56 – citations retrieved) April 2022 |
| ***WANFANG (via WANFANG DATA)*** | （题名或关键词:(Compliance OR Adherence OR 依从性 OR 佩戴时长)）and（题名或关键词:(脊柱侧弯 OR 特发性脊柱侧弯 OR scoliosis )）  (63 – citations retrieved) April 2022 |
