# Supplementary material for: Which interventions may improve bracing compliance in adolescent idiopathic scoliosis? A systematic review and meta-analysis
Source: PLoS One. 2022 Jul 20;17(7):e0271612. doi: 10.1371/journal.pone.0271612 (PMC9299303; doi:10.1371/journal.pone.0271612)
Supplement: S3 Table — (DOCX) [file pone.0271612.s003.docx]

**S3 Table List of excluded studies and reasons for exclusion.**

| **Study** | **Reason for exclusion** |
| --- | --- |
| Crostelli 2017[1] | No measure of compliance outcomes |
| deMauroy 2015[2] | No measure of compliance outcomes |
| Donzelli 2012[3] | No comparable control group, all participants received intervention |
| Hasler 2010[4] | No comparable control group, all participants received intervention |
| Law 2017[5] | No comparable control group, all participants received intervention |
| Rivett 2009[6] | Observational study without an intervention method |
| Schiller 2010[7] | Review article |
| Schwieger 2017[8] | Observational study without an intervention method |

1. D. CMMOMMM. ART brace in idiopathic scoliosis treatment. European Spine Journal. 2017;26:1388.

2. de Mauroy JC, Journe A, Gagaliano F, Lecante C, Barral F, Pourret SJS. The new Lyon ARTbrace versus the historical Lyon brace: a prospective case series of 148 consecutive scoliosis with short time results after 1 year compared with a historical retrospective case series of 100 consecutive scoliosis; SOSORT award 2015 winner. 2015;10(1):26.

3. Donzelli S, Zaina F, Negrini SJS. It is possible to make patients use braces the hours prescribed: first results from the thermobrace clinical everyday usage. 2012;7(1):O29.

4. Hasler CC, Wietlisbach S, Büchler PJJocso. Objective compliance of adolescent girls with idiopathic scoliosis in a dynamic SpineCor brace. 2010;4(3):211-8.

5. Lou E, Hill D, Raso J, Mahood J, Moreau MJSiht, informatics. Improving brace wear with active brace system. 2006;123:498-504.

6. Rivett L, Rothberg A, Stewart A, Berkowitz R. The relationship between quality of life and compliance to a brace protocol in adolescents with idiopathic scoliosis: a comparative study. BMC musculoskeletal disorders. 2009;10:5.

7. Schiller JR, Thakur NA, Eberson CP. Brace management in adolescent idiopathic scoliosis. Clinical orthopaedics and related research. 2010;468(3):670-8.

8. Schwieger T, Campo S, Weinstein SL, Dolan LA, Ashida S, Steuber KRJJopo. Body image and quality of life and brace wear adherence in females with adolescent idiopathic scoliosis. 2017;37(8):e519.
