# Supplementary material for: Which interventions may improve bracing compliance in adolescent idiopathic scoliosis? A systematic review and meta-analysis
Source: PLoS One. 2022 Jul 20;17(7):e0271612. doi: 10.1371/journal.pone.0271612 (PMC9299303; doi:10.1371/journal.pone.0271612)
Supplement: S4 Table — (DOCX) [file pone.0271612.s004.docx]

**S4 Table Overall GRADE quality assessment for primary outcome (bracing compliance).**

| Studies (n) | Study design | Risk of bias | Inconsistency | Indirectness | Imprecision | Publication bias | Quality |
| --- | --- | --- | --- | --- | --- | --- | --- |
| Comparison: sensor monitoring vs non-monitoring | | | | | | | |
| 2 | RCT | Serious | Not serious | Not serious | Not serious | Strongly suspected | ⨁⨁◯◯  Low |
| Comparison: complete team approach vs non-complete or no team approach | | | | | | | |
| 1 | case–control study | Serious | Serious | Not serious | Serious | Strongly suspected | ⨁◯◯◯  Very low |
| Comparison: outpatient service vs hospitalization (at initial stage of bracing treatment) | | | | | | | |
| 1 | Controlled clinical trial | Serious | Serious | Not serious | Serious | Strongly suspected | ⨁◯◯◯  Very low |
| Comparison: Cognitive Behavioral Approach (CBA) + Physiotherapic Scoliosis Specific Exercises (PSSE) vs no intervention | | | | | | | |
| 1 | Retrospective controlled cohort study | Serious | Serious | Not serious | Not serious | Strongly suspected | ⨁◯◯◯  Very low |
| Comparison: Automated pressure-adjustable orthosis (PO) vs Conventional rigid orthosis (CO) | | | | | | | |
| 1 | RCT | Serious | Not serious | Not serious | Serious | Strongly suspected | ⨁⨁◯◯  Low |

GRADE Working Group grades of evidence

**High quality**: Further research is very unlikely to change our confidence in the estimate of effect.

**Moderate quality**: Further research is likely to have an important impact on our confidence in the estimate of effect and may change the estimate.

**Low quality**: Further research is very likely to have an important impact on our confidence in the estimate of effect and is likely to change the estimate.

**Very low quality**: We are very uncertain about the estimate.
